# Supplementary material for: Deletion of wfs1 Impairs Oligodendrocyte Precursor Cells Dorsal Distribution and Myelination Through the wfs1-hmgcs1 Axis in Zebrafish
Source: Biology (Basel). 2026 Aug 16;15(16):1402. doi: 10.3390/biology15161402 (PMC13510151; doi:10.3390/biology15161402)
Supplement: Supplementary file 1 [file biology-15-01402-s001.zip › File S1-Transcriptome sequencing data and differentially expressed genes (DEGs) in wfs1-- zebrafish at 5 dpf/2.KEGG/wfs1KOvsWT/ALL/src/dre04068.html]

dre04068


- dre:406473

- Up regulated genes

406473(3.55746093515464)

- dre:100000252

- Up regulated genes

100000252\_3(1.94434154854557) 100000252(1.09163460881879)

- Down regulated genes

100000252\_1(-1.7718817168434)

- dre:571431

- Down regulated genes

571431(-6.42862549702529)

- dre:563708

- Down regulated genes

563708(-3.59233336704936)

- dre:566841

- Up regulated genes

566841(1.91411798034227)

- dre:245702

- Up regulated genes

245702(1.27755220735994)

- Down regulated genes

245702\_1(-1.45433245048201)

- dre:767751

- Up regulated genes

767751\_1(3.05541031760882)

- dre:565576

- Up regulated genes

565576(3.58116359058899)
- dre:280649

- Up regulated genes

280649\_1(2.27023747569103)

- dre:431763

- Up regulated genes

431763(1.93865999132)

- Down regulated genes

431763\_1(-4.20440659354098)

- dre:431763

- Up regulated genes

431763(1.93865999132)

- Down regulated genes

431763\_1(-4.20440659354098)

- dre:117508

- Up regulated genes

117508\_1(1.34887174636376) 117508(1.41697225185209)

- dre:100006858

- Down regulated genes

100006858\_1(-1.49071226984431)

- dre:100000242

- Down regulated genes

100000242(-6.34512572021214)
- dre:326974

- Up regulated genes

326974\_1(1.07837684837292)

- dre:324140

- Up regulated genes

324140\_1(4.93450118149538)

- Down regulated genes

324140(-5.90052475401947)

- dre:562552

- Up regulated genes

562552(1.50682290794657)

- dre:559723

- Up regulated genes

559723(3.63719210583924)

Close
